# Modeling dose-dependent temperature responses to methamphetamine

**DOI:** 10.1186/1471-2202-13-S1-P50

**Published:** 2012-07-16

**Authors:** Yaroslav Molkov, Dmitry Zaretsky, Maria Zaretskaia, Dan Rusyniak

**Affiliations:** 1 Department of Mathematical Sciences, Indiana University – Purdue University Indianapolis, IN 46202, USA; 2 Department of Emergency Medicine, Indiana University School of Medicine, Indianapolis, IN 46202, USA

## 

Amphetamine derivatives are among the most abused drugs in the world. Long-term use can lead to cognitive, neurophysiological, and neuroanatomical deficits. These problems are enhanced by hyperthermia, which itself is a major mortality factor in drug abusers. Temperature responses to amphetamine injections are multiphasic including both hypothermic and hyperthermic phases. These responses are highly dependent on ambient temperature, previous exposure to the drug, and the involvement of various brain areas. It is therefore not surprising that most amphetamine research is performed with only one dose and/or only at one ambient temperature – neither of which may adequately predict real-life situations.

**Figure 1:**
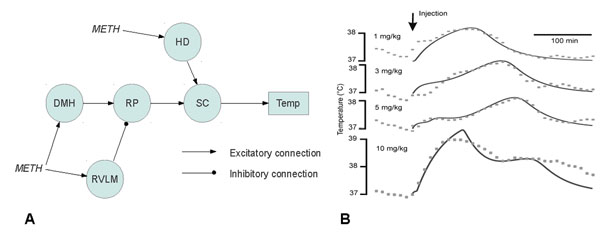
**A.** Neurocircuitry involved in neural control of temperature: DMH – Dorsomedial Hypothalamus; RP – Raphe Pallidus; RVLM – Rostral Ventrolateral Medulla; SC – spinal cord; HD – “high dose” activator; Temp – body temperature. Arrows show sites affected by methamphetamine (*METH*). **B.** Dose-dependent temperature responses to a single injection of *METH*: gray dots – experiment, black solid lines – as produced by the model.

In this study we have attempted to mathematically describe temperature responses to several doses of methamphetamine (*METH*); drugs were intraperitoneally injected in freely moving rats (N=3-4 per dose) at room temperature. Our experimental data are consistent with earlier published data [1]: intermediate dose of *METH* (5 mg/kg) causes less hyperthermia than both low (1 mg/kg) and high (10 mg/kg) doses of the drug. Also, maxima of all responses have different latency: responses to low and high doses are virtually immediate, while response to an intermediate dose is delayed. Based on the presumed neurocircuitry (Fig.1A), we have devised a mathematical model in the form of an artificial neural network with first order pharmacokinetics. Each population was described as a three layer perceptron. All model parameters (weights of connections, sensitivity to the drug, pharmacokinetics and temperature response time constants) were subject to fitting three time-series (temperature responses to 1, 5, and 10 mg/kg of *METH* obtained in the experiment, Fig.1B). This model appeared to be sufficient to explain such complex phenomenon as dose-dependence of the temperature response (see Fig.1B), and reasonably predicted the effect of an additional dose *METH* not used in constructing the model (D=3 mg/kg on Fig.1B).

Our results are a promising start to constructing a comprehensive and physiologically relevant model of temperature responses to derivatives of amphetamine. Future will experimentally confirm the identity of the specific brain areas of the interconnected nodes. The power of mathematically modeling is that along with predicting responses to amphetamines in laboratory conditions it may predict scenarios in which life-threatening hyperthermia can occur.
